# Ubiquitin-specific protease 1 facilitates tumor immune escape from natural killer cells and predicts the prognosis in small cell lung cancer

**DOI:** 10.32604/or.2024.046895

**Published:** 2024-12-20

**Authors:** SHIQIN JIANG, YICHUN TANG, FENG MA, YUCHUN NIU, LEI SUN

**Affiliations:** 1 Department of Clinical Pharmacy, Shenzhen Hospital of Integrated Traditional Chinese and Western Medicine, Shenzhen, 518104, China; 2 Department of Pathology, Zhujiang Hospital, Southern Medical University, Guangzhou, 510280, China; 3 Department of Radiation Oncology, The First People’s Hospital of Foshan, Foshan, 528000, China; 4 Department of Oncology, The First Affiliated Hospital of Jinan University, Jinan University, Guangzhou, 510630, China; 5 Department of Oncology, The First Dongguan Affiliated Hospital, Guangdong Medical University, Dongguan, 523721, China

**Keywords:** Ubiquitin-specific protease 1 (USP1), Natural killer (NK) cell, Small cell lung cancer (SCLC), Prognosis, Immune escape

## Abstract

**Objective:**

Small cell lung cancer (SCLC) is commonly recognized as the most fatal lung cancer type. Despite substantial advances in immune checkpoint blockade therapies for treating solid cancers, their benefits are limited to a minority of patients with SCLC. In the present study, novel indicators for predicting the outcomes and molecular targets for SCLC treatment were elucidated.

**Methods:**

We conducted bioinformatics analysis to identify the key genes associated with tumor-infiltrating lymphocytes in SCLC. The functional role of the key gene identified in SCLC was determined both *in vitro* and *in vivo*.

**Results:**

A significant correlation was observed between patient survival and CD56dim natural killer (NK) cell proportion. Furthermore, we noted that the hub gene ubiquitin-specific protease 1 (USP1) is closely correlated with both CD56dim NK cells and overall survival in SCLC. Bioinformatics analysis revealed that USP1 is upregulated in SCLC. In addition, gene set enrichment analysis revealed that USP1 overexpression hinders NK cell-mediated immune responses. By co-cultivating NK-92 cells with SCLC cells, we demonstrated that NK cell cytotoxicity against SCLC could be improved either via USP1 knock-down or pharmacological inhibition. Furthermore, using a nude-mice xenograft tumor model, we noted that USP1 inhibition effectively suppressed tumor proliferation and increased the expression of NK cell-associated markers.

**Conclusions:**

Our study findings highlight the importance of NK cells in regulating SCLC. USP1 overexpression can inhibit NK cell-mediated immunity; therefore, USP1 may serve not only as a prognostic biomarker but also as a potential molecular target of SCLC therapy.

## Introduction

Small cell lung cancer (SCLC) is considered the most fatal type of lung cancer; it accounts for approximately 15% of all lung cancers [1]. Although most patients with SCLC initially respond positively to cytotoxic chemotherapy, they frequently develop resistance over time, resulting in a low 5-year survival rate of <7% [2,3]. Despite the application of immune checkpoint blockade (ICB) to platinum-based chemotherapy, there has been limited progress in patient survival, with a large proportion of SCLC patients not responding to ICBs [4,5]. To enhance the effectiveness of therapeutic approaches, it is crucial to further elucidate the patterns of immune infiltration and the interactions between the immune system and tumors in SCLC.

The goal of ICB therapy is to enhance T-cell-mediated responses by targeting T-cell checkpoints [6]. The insensitivity of patients with SCLC to these T-cell-based immunotherapies has increased the interest of researchers in other cytotoxic immune cells, including natural killer (NK) cells [7,8]. In the present study, a comprehensive analysis was conducted to elucidate the correlation between immune infiltration and the overall survival of individuals with SCLC from two previously published cohorts [9,10]. We observed a significant association between patients survival and CD56^dim^ NK cells. Ubiquitin-specific protease 1 (USP1) was identified as a hub gene closely associated with these cells.

USP1 is an important deubiquitinating enzyme that plays a vital role in various cellular and biological processes, including genome integrity maintenance, cell cycle regulation, migration, and cellular homeostasis [11–13]. Recent evidence suggests the vital role of USP1 in both innate and adaptive immunities; however, its specific role in regulating the immune system remains unclear [14,15]. Furthermore, USP1 may contribute to cancer development and metastasis [16,17]. In addition, studies have revealed that increased USP1 expression is associated with a poorer prognosis in different cancer types [18–20]. Nevertheless, the role of USP1 in SCLC remains unclear.

In the present study, we found that USP1 was highly expressed in SCLC and associated with poorer outcomes. Furthermore, we have demonstrated that increased levels of USP1 can hinder the immune responses of NK cells in laboratory settings. Therefore, USP1 can serve as a promising prognostic factor and therapeutic target in SCLC.

## Materials and Methods

### Data collection

RNA sequencing data and the clinical information of two publicly available cohorts, namely, the George’ [9] and GSE60052 [10] cohorts, were used to determine the correlation between tumor-infiltrating lymphocyte (TILs) and the overall survival of patients with SCLC. The George’s cohort consists of 81 patients, while GSE60052 includes 79 patients. In addition, these two datasets were used to identify the differentially expressed genes (DEGs) between the high- and low-USP1 expression groups.

To identify the DEGs between cancer and normal samples, the microarray dataset GSE149507 [21] was used; this dataset comprises 18 paired SCLC and normal samples. Furthermore, the datasets from the Oncomine database (https://www.oncomine.org) and Cancer Cell Line Encyclopedia (CCLE) database (https://portals.broadinstitute.org/ccle/) were used to validate USP1 expression.

### Immune infiltration analysis

Single-sample gene set enrichment analysis (GSEA) [22] of the George’s et al. [9] and GSE60052 [10] cohorts was performed to elucidate the relative abundance of 28 TILs. The marker genes for immune cells were obtained from Bindea et al. [23]. The following TILs were identified in this analysis: activated B cell, memory B cells, immature B cells, activated CD4 T cell, effector memory CD4^+^ T cells, central memory CD4^+^ T cells, activated CD8 T cell, central memory CD8^+^ T cells, effector memory CD8^+^ T cells, activated dendritic cell, immature dendritic cells, NK cells, CD56^bright^ NK cell, CD56^dim^ NK cell, eosinophil, gamma delta T cell, NK T cells, regulatory T cells, T follicular helper cells, type 1 T helper cells, type 17 T helper cells, type 2 T helper cells, macrophage, mast cell, myeloid-derived suppressor cell, monocyte, neutrophil and plasmacytoid dendritic cell.

### DEG analysis

DEGs in various subgroups were identified using the limma R package. The *p*-values were adjusted using the Benjamini-Hochberg false discovery rate correction to account for multiple testing. We used the following thresholds to determine differentially expressed genes (DEGs): an absolute log2 fold change greater than 1.0 and a false discovery rate adjusted *p*-value less than 0.05. The SCLC samples from the George’s [9] and GSE60052 [10] cohorts were analyzed by comparing samples with a high proportion of CD56^dim^ NK cells with those with a low proportion. Furthermore, the GSE149507 cohort [21] was used to identify the DEGs between tumor and normal samples. Venn analysis was performed to identify the common DEGs. Moreover, DEG analysis was performed by comparing high-USP1 expression samples with low-USP1 expression samples in the George’s and GSE60052 cohort.

### GSEA

The biological functions of USP1 were annotated via GSEA [22] using the R package clusterProfiler. A gene set was considered to be significantly enriched if the adjusted *p*-value was <0.05. The enrichment analyses for Kyoto Encyclopedia of Genes and Genomes (KEGG) pathways and Gene Ontology (GO) terms were carried out using clusterProfiler.

### Cell culture

The NCI-H446 SCLC cell line was purchased from the American Type Culture Collection (USA). The mycoplasma testing of the cell lines have conducted the tests, and the results were negative. All the cell lines used in our experiments are mycoplasma-free to guarantee the accuracy and reliability of the data. The cells were cultured in RPMI-1640 medium (Gibco, USA) supplemented with 10% fetal bovine serum (Gibco) in a humidified incubator at 37°C with 5% CO_2_. The Department of Hepatobiliary Surgery at Zhujiang Hospital of Southern Medical University kindly provided NK-92 cells. The cells were purchased from the Chinese National Collection of Authenticated Cell Cultures and were cultured in RPMI-1640 medium supplemented with stable L-glutamine, 10% Fetal Bovine Serum, 100 U/mL penicillin, 100 mg/mL streptomycin, and 200 IU/mL IL-2 (Cellcook, Guangzhou, China).

### Cell transfection

To knock down USP1, Lipofectamine 3000 (Thermo Scientific, USA) was used according to the manufacturer’s instructions to transiently transfect the cells with a siRNA against USP1 (GenePharma, Shanghai, China). The siRNA sequences were as follows: USP1 siRNA-1, sense (5′-3′) GGAUUUCACAGAUUCUCAATT, antisense (5′-3′) UUGAGAAUCUGUGAAAUCCTT; and USP1 siRNA-2, sense (5′-3′) GGUUAAAGUCUGCAACUAATT, antisense (5′-3′) UUAGUUGCAGACUUUAACCTT.

### Quantitative real-time polymerase chain reaction (qRT-PCR)

TRIzol reagent (Invitrogen, USA) was used to extract total RNA from the cells. Subsequently, cDNA was synthesized using 2 μg of RNA and the PrimeScript RT reagent kit (Tiangen, Beijing, China), following the manufacturer’s instructions. The ABI Illumina instrument (Foster, USA) with SYBR Green (Tiangen) was used to perform qRT-PCR. The PrimeScript RT reagent kit (Tiangen, Beijing, China) was used to perform reverse transcription. Gene expression was measured using the relative quantification (2^−ΔΔCt^) method and normalized to GAPDH. The primer sequences were as follows:

USP1: F 5′-CTTGTTACCATTTGTGGGACTG-3′, R5′-ATTGGCTTCATCCTTTAGAGCT-3′.

GAPDH: F 5′-AAATCAAGTGGGGCGATGCT-3′, R5′-CAAATGAGCCCCAGCCTTCT-3′.

### Western blotting

RIPA buffer (Thermo Scientific) was used to lyse the cells. The bicinchoninic acid (BCA) protein assay kit (Beyotime, Shanghai, China) based on the BCA method was used to measure protein concentrations. Then, the cell protein lysates were electrophoresed on 10% SDS-polyacrylamide gels (Solarbio, beijing, China), transferred to polyvinylidene fluoride membranes (Solarbio) and incubated with primary and secondary antibodies. GAPDH served as the loading control. Anti-GAPDH (ab8227) was purchased from Abcam (UK) and anti-USP1 (DF2257) was purchased from Affinity Biosciences (USA). The antibody dilution ratio in 1:1000.

### Immunofluorescence analysis

Cells were seeded into 24-well plates and incubated for 24 h, with different concentrations of ML323 (Selleck, China). Then, the cells were washed three times with PBS and fixed with 4% paraformaldehyde (Solarbio). After permeabilization with 0.1% Triton X-100 (Solarbio) for 10 min, the cells were blocked with PBS plus 1% BSA for 1 h. Cells were then incubated with the anti-USP1 primary antibody (DF2257, Affinity) overnight at 4°C. After washing three times with PBS, the cells were incubated with a fluorescent secondary antibody (Alexa Fluor 488-conjugated anti-rabbit antibody, Thermo Scientific) at room temperature for 1 h. Then, 4′,6-Diamidino-2-phenylindole (Sigma, USA) was used to stain the nucleus. A confocal microscope (Nikon, ECLIPSE Ti2-E) with a 63× oil objective lens was used to capture the images.

### Lactate dehydrogenase (LDH)-release assay

The LDH release assay was performed to determine the inhibitory effect of USP1 on NK cell cytotoxicity in SCLC cells. Briefly, NCI-H446 cells transfected with either siRNA-USP1 or control siRNA were seeded into 96-well plates at a density of 3 × 10^4^ cells/well and incubated for 24 h. Then, NCI-H446 cells were cocultured with different densities of NK-92 cells (described as effector to target ratio (E/T ratio) = 25:1, 5:1 and 1:1). After coculturing for 4 h, cell-free supernatants were harvested. The LDH levels in the supernatants, released by damaged SCLC cells, were determined using an LDH cytotoxicity assay kit (Beyotime, China) following the manufacturer’s instructions. Absorbance readings were taken at 490 nm using a microplate reader (Spectra Max iD5).

### Flow cytometry analysis

First, NCI-H446 cells were labeled with calcein violet AM (Invitrogen) and then cocultured with NK-92 cells at a ratio of 1:2 (target:effector) for 6 h. Thereafter, cells were harvested using EDTA-free trypsin (Gibco), washed three times with PBS, and resuspended in a binding buffer. Cells were incubated with propidium iodide (Invitrogen) and annexin V–fluorescein isothiocyanate (Beibo, China) in the dark for 10 min, followed by measurements in a flow cytometer (Beckman CytoFLEX). Annexin V-stained cells were considered apoptotic cells. The results were expressed as the percentage of apoptotic cells in total cells.

### Tumor xenograft experiments

The Ethics of Animal Experiments of Guangdong Medical University (Ethical number: GDY2302565) approved all animal experiments. The Declaration of Helsinki was strictly followed. Ten 3–4-week-old male BALB/c nude mice were purchased from Guangdong Experimental Animal Center and housed in a SPF-grade animal room; the mice were randomly divided into two groups. Mice were subcutaneously injected with 1 × 10^7^ tumor cells (NCI-H446) suspended in 150 µL PBS. On day 9 after implantation, the tumors became palpable, with a diameter of approximately 5 mm. Then, the USP1 inhibitor ML323 [19,24] (Selleck, USA) or vehicle solution was intraperitoneally injected (days 10–21, every day) into mice. After tumor formation, tumor size was monitored every 3 days. Four weeks after injection, all mice were euthanized under general anesthesia. The following formula was used to calculate tumor volume: (length × width^2^)/2.

### Statistical analysis

R version 4.0.0 was used to perform statistical computations. Univariate and multivariate Cox regression models were used to determine the factors independently contributing to overall survival. For multivariate Cox regression analysis, we considered several potential confounding variables, including age, sex, smoking history, and clinical stage as covariates in our analysis. Survival curves were generated using the Kaplan-Meier method and compared statistically using the log-rank test. Comparisons between groups were performed using the unpaired *t-*test or Mann-Whitney U test. The two-tailed approach was used to conduct all statistical tests. A *p*-value of <0.05 was considered statistically significant. The statistical significance was described as follows: ns, not significant; *p < 0.05; **p ≤ 0.01; ***p ≤ 0.001; ****p ≤ 0.0001.

## Results

### CD56^dim^ NK cell abundance was an independent prognostic factor for patients with SCLC

**Figure 1:**
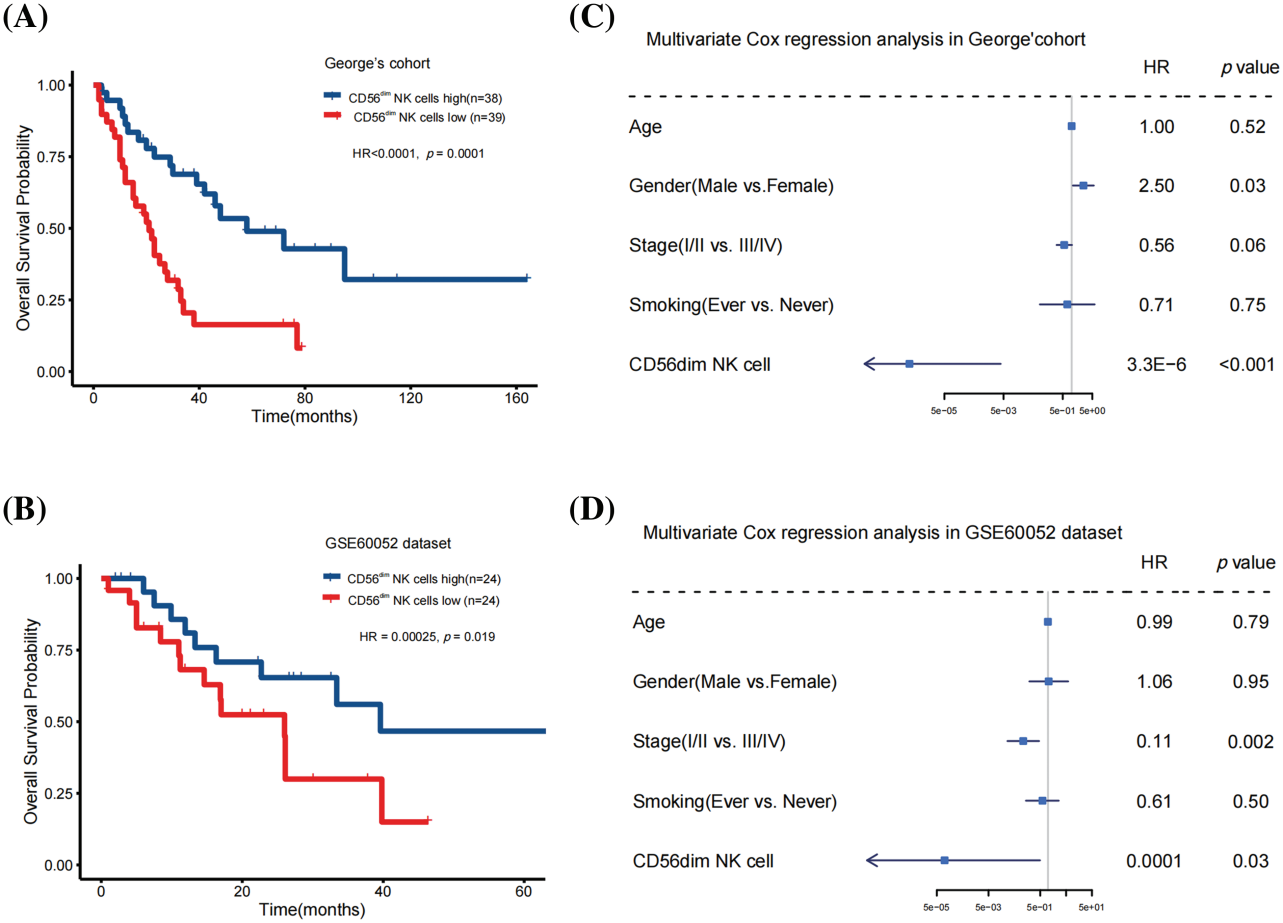
CD56^dim^ NK cell abundance was an independent prognostic factor for patients with SCLC. (A) Kaplan-Meier curves depicting the overall survival in the George’s cohort (Log-rank test). (B) Kaplan-Meier curves depicting the overall survival in the GSE60052 cohort (Log-rank test). (C) Multivariate Cox regression analysis conducted on the George’s cohort to analyze the impact of CD56^dim^ NK cell abundance on overall survival. (D) Multivariate Cox regression analysis conducted on the GSE60052 cohort to analyze the impact of CD56^dim^ NK cell abundance on overall survival.

We investigated 28 types of TILs in SCLC samples from George’s cohort [9] and the GSE60052 cohort [10] using the single-sample GSEA algorithm. The correlation between TILs and prognosis was analyzed using univariate Cox regression analysis and KM survival analysis. Only CD56^dim^ NK cell abundance was significantly associated with patient survival (George’s cohort: hazard ratio = 8.90E-07 [95% confidence interval, 7.6E-10 to 0.001], *p* = 0.0001; GSE60052 cohort: hazard ratio = 0.00025 [95% confidence interval, 2.4E-7 to 0.26], *p* = 0.019; Figs. 1A and 1B and Suppl. Table 1). Furthermore, multivariate analysis revealed that even after adjusting for age, gender, smoking and clinical stage, CD56^dim^ NK cell abundance positively correlated with overall survival in both cohorts (George’s cohort: hazard ratio = 3.3E-6 [95% confidence interval, 2.7E-9 to 0.004], *p* < 0.001; GSE60052 cohort: 0.0001 [95% confidence interval, 2.1E-08 to 0.49], *p* = 0.03; Figs. 1C and 1D).

### USP1 was identified as the hub gene correlated with CD56^dim^ NK cells

**Figure 2:**
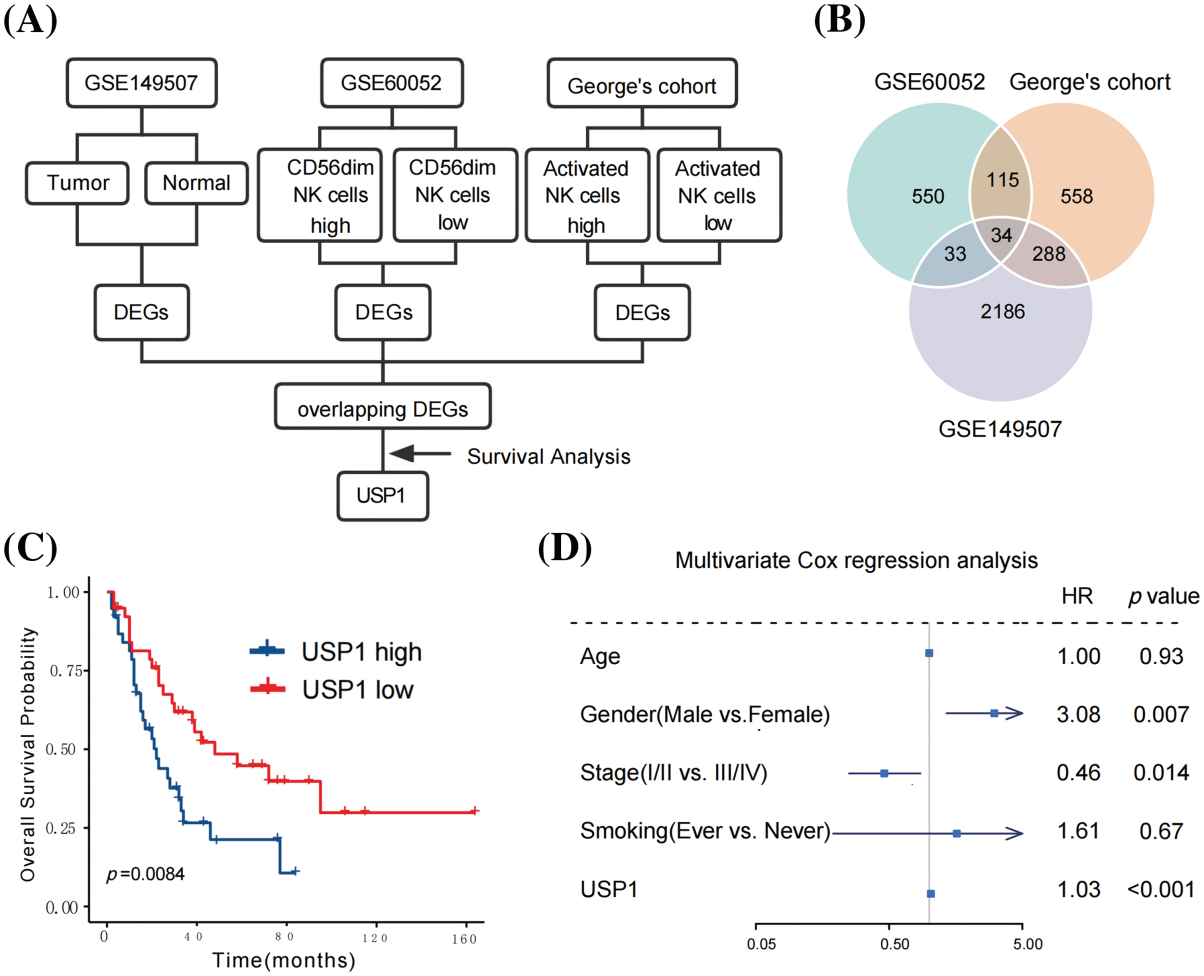
USP1 was identified as the hub gene correlated with CD56^dim^ NK cells. (A) The flowchart for hub gene identification. (B) Venn diagram of hub genes identification. (C) USP1 expression was notably correlated to overall survival (Log-rank test). (D) Multivariate Cox regression analysis revealed that USP1 gene expression was an independent prognostic factor for overall survival.

Fig. 2A illustrates a flowchart for hub gene identification. First, the DEGs between high- and low-CD56^dim^ NK cell samples were analyzed to identify the hub gene associated with CD56^dim^ NK cells in SCLC. In George’s cohort [9], 691 upregulated genes and 304 downregulated genes were identified in high-CD56^dim^ NK cell samples (Fig. 2B). Similarly, 650 upregulated genes and 82 downregulated genes were identified in the GSE60052 cohort [10] (Fig. 2B). Next, the differential gene expression of 18 paired SCLC tumor and adjacent lung tissues from the GSE149507 [21] cohort was analyzed; 2542 DEGs, with 1136 upregulated genes and 1406 downregulated genes were identified (Fig. 2B). Finally, 34 overlapping DEGs were identified (Fig. 2B).

Seventy-seven patients with survival data from George’s cohort [9] were subjected to survival analysis to determine the potential prognostic value of the 34 candidate genes. Among the identified genes, only USP1 was notably correlated with overall survival (Fig. 2C). To further assess the robustness and reliability of this association, we performed a sensitivity analysis by comparing the prognostic significance of USP1 expression at different cutoff values. The sensitivity analysis, presented in Suppl. Table 2, consistently demonstrated significant associations between USP1 expression and overall survival across various cutoff values. Furthermore, multivariate Cox regression analysis revealed that USP1 gene expression was an independent prognostic factor for overall survival; the hazard ratio and 95% confidence interval were 1.03 and 1.03–3.60, respectively (*p* < 0.001, Fig. 2D). Subsequently, USP1 was identified as the key gene in SCLC.

### USP1 overexpression inhibited NK cell-mediated immunity

**Figure 3:**
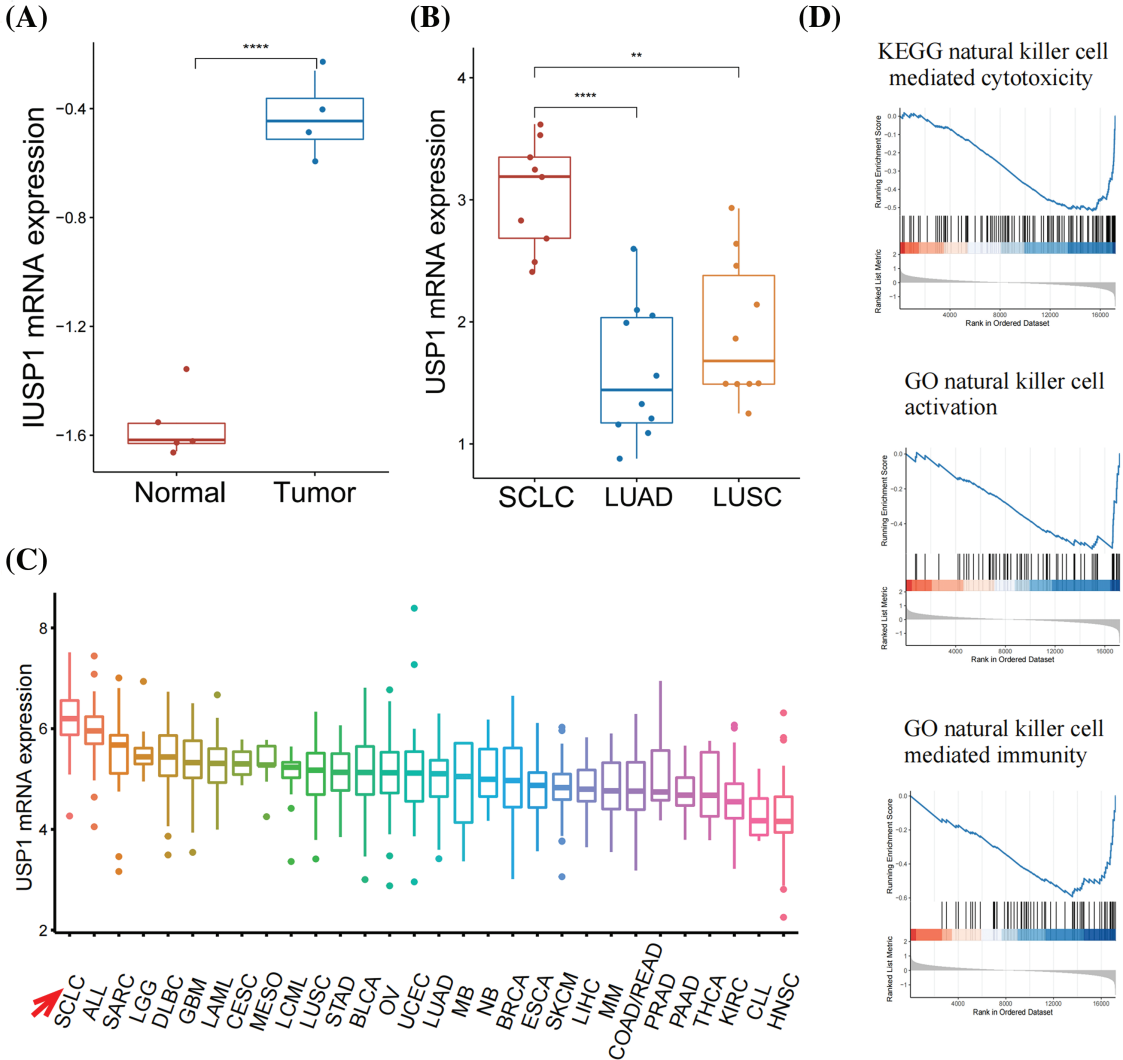
USP1 overexpression inhibited NK cell-mediated immunity. (A) USP1 expression was compared between tumor and normal samples in the GSE149507 cohort. The statistical significance was determined using the Mann-Whitney U test. ****p ≤ 0.0001. (B) USP1 expression was compared between SCLC, NSCLC, and normal lung samples in Oncomine database. The statistical significance was determined using the Mann-Whitney U test. **p ≤ 0.01; ****p ≤ 0.0001. (C) The expression of USP1 was evaluated in various types of cancer cell lines from the CCLE database. (D) GSEA analysis was performed to analyze NK cell-related pathways.

We compared USP1 expression in tumor and normal samples from the GSE149507 cohort [21]. USP1 was significantly expressed in the tumor samples (Fig. 3A). Furthermore, using the Oncomine database, we observed that USP1 expression was markedly increased in SCLC tissues than in Non-SCLC and normal lung tissues (Fig. 3B). Moreover, using the CCLE database, we observed that USP1 expression was the most elevated in SCLC cell lines compared to any other type of cancer cell lines (Fig. 3C).

**Figure S1:**
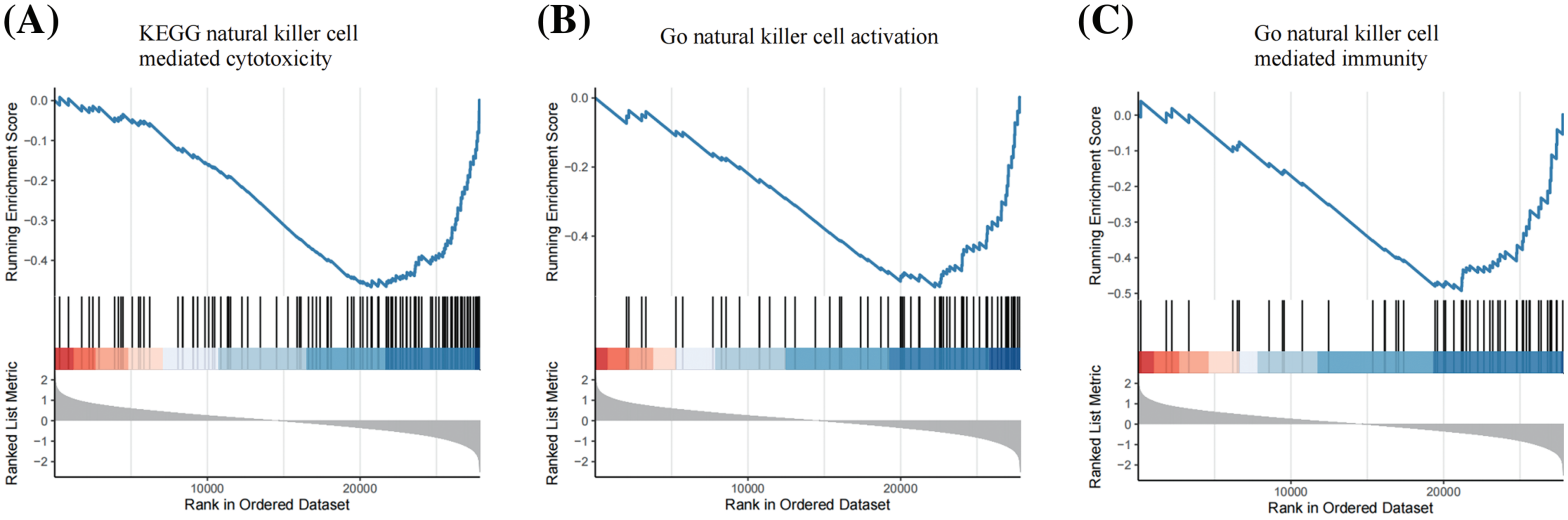
GSEA revealed that USP1 was negatively associated with NK cell related pathways, including KEGG natural killer cell mediated cytotoxicity (A), Go natural killer cell activation (B) and Go natural killer cell mediated immunity (C).

GSEA was performed to annotate the biological functions of USP1. We noted the negative association between USP1 and several NK cell-related pathways, including NK cell-mediated cytotoxicity, NK cell activation, and NK cell-mediated immunity (Fig. 3D and Suppl. Fig. 1).

### USP1 inhibition enhanced SCLC sensitivity to NK cell cytotoxicity

**Figure 4:**
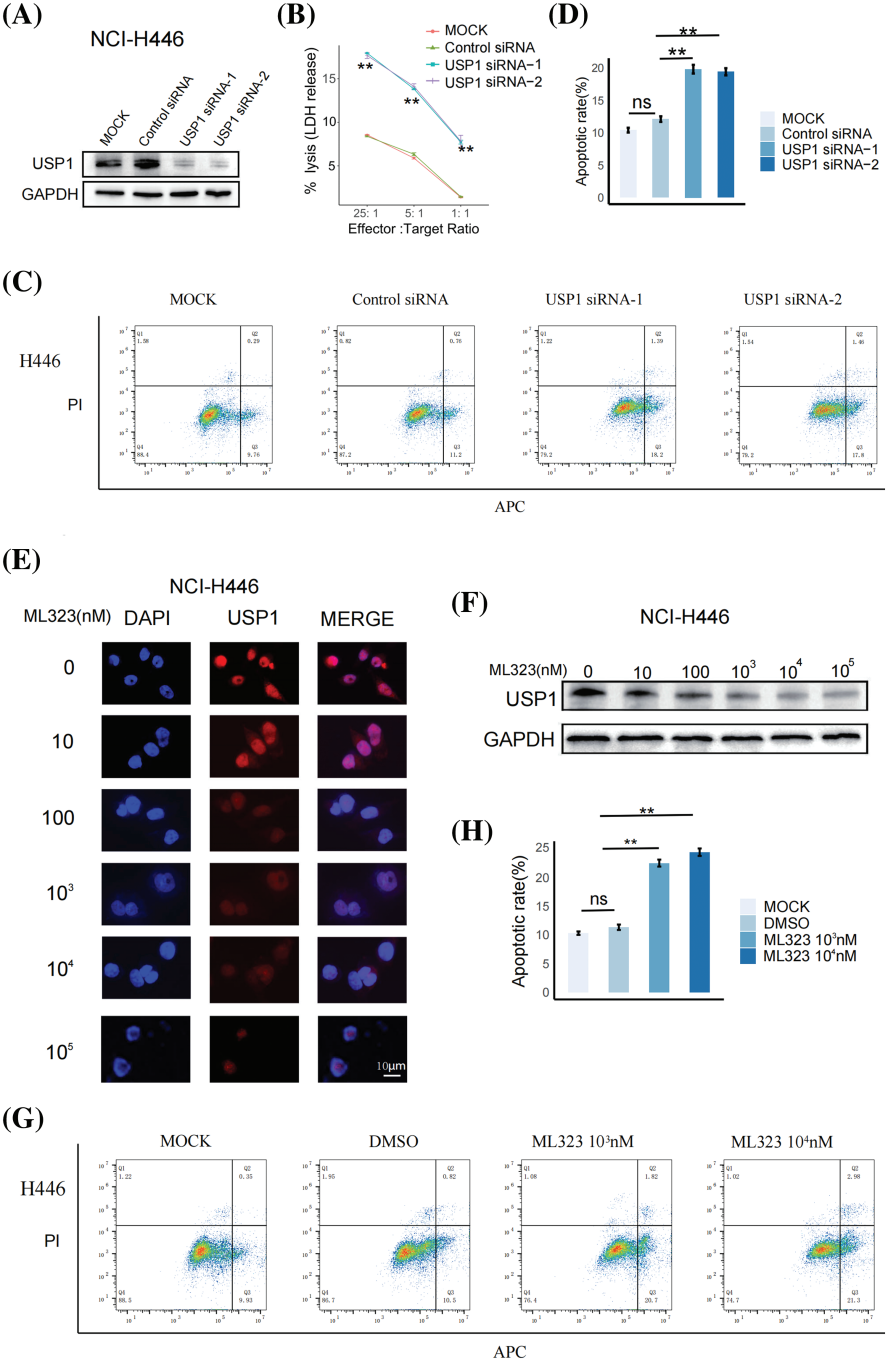
USP1 inhibition enhanced SCLC sensitivity to NK cell cytotoxicity. (A) Western blotting was performed to assess USP1 expression in NCI-H446 cells following siRNA transfection. (B) The cytotoxicity of NK-92 cells against NCI-H446 cells was measured using LDH assay at different effector–target (E:T) ratios. The unpaired *t*-test was used to perform statistical analysis (**p < 0.01). (C) Flow cytometry was employed to detect apoptosis in NCI-H446 cells co-cultured with NK-92 cells after siRNA transfection. (D) The apoptosis rate was calculated using the mean and standard deviation. The unpaired *t*-test was used to perform statistical analysis (ns, not significant; **p < 0.01). (E) Immunofluorescence assay was conducted on NCI-H446 cells after exposure to different doses of ML323 (Scale bar, 10 μm). (F) Western blotting was performed to assess the expression of USP1. (G) Flow cytometry was utilized for apoptosis detection in NCI-H446 cells co-cultured with NK-92 cells after treatment with ML323. (H) The apoptosis rate was calculated using the mean and standard deviation. The unpaired *t*-test was used to perform statistical analysis (ns, not significant; **p < 0.01).

To determine the effect of USP1 on NK cell cytotoxicity against SCLC, we downregulated USP1 expression in NCI-H446 cells via siRNA transfection and cocultured the cells with NK-92 cells. Then, western blotting was performed to assess the changes in USP1 protein levels (Fig. 4A). The LDH assay revealed that USP1 knockdown significantly enhanced NK cell cytotoxicity against SCLC cells (Fig. 4B). Furthermore, flow cytometry analysis revealed a notable increase in apoptosis levels when USP1 expression was downregulated, in contrast to the control group (Figs. 4C and 4D).

We next investigated whether pharmacological inhibition of USP1 using ML323, a selective USP1 inhibitor, can enhance NK-cell cytotoxicity against SCLC. NCI-H446 cells were treated with different concentrations of ML323 for 24 h. Immunofluorescence analysis and western blotting revealed that ML323 significantly decreased USP1 expression (Figs. 4E and 4F). Subsequently, the apoptosis of NCI-H446 cells cocultured with NK92 cells was assessed via flow cytometry. There was an increase in the apoptosis of ML323-treated SCLC cells (Figs. 4G and 4H).

### USP1 inhibition suppressed SCLC growth in vivo

**Figure 5:**
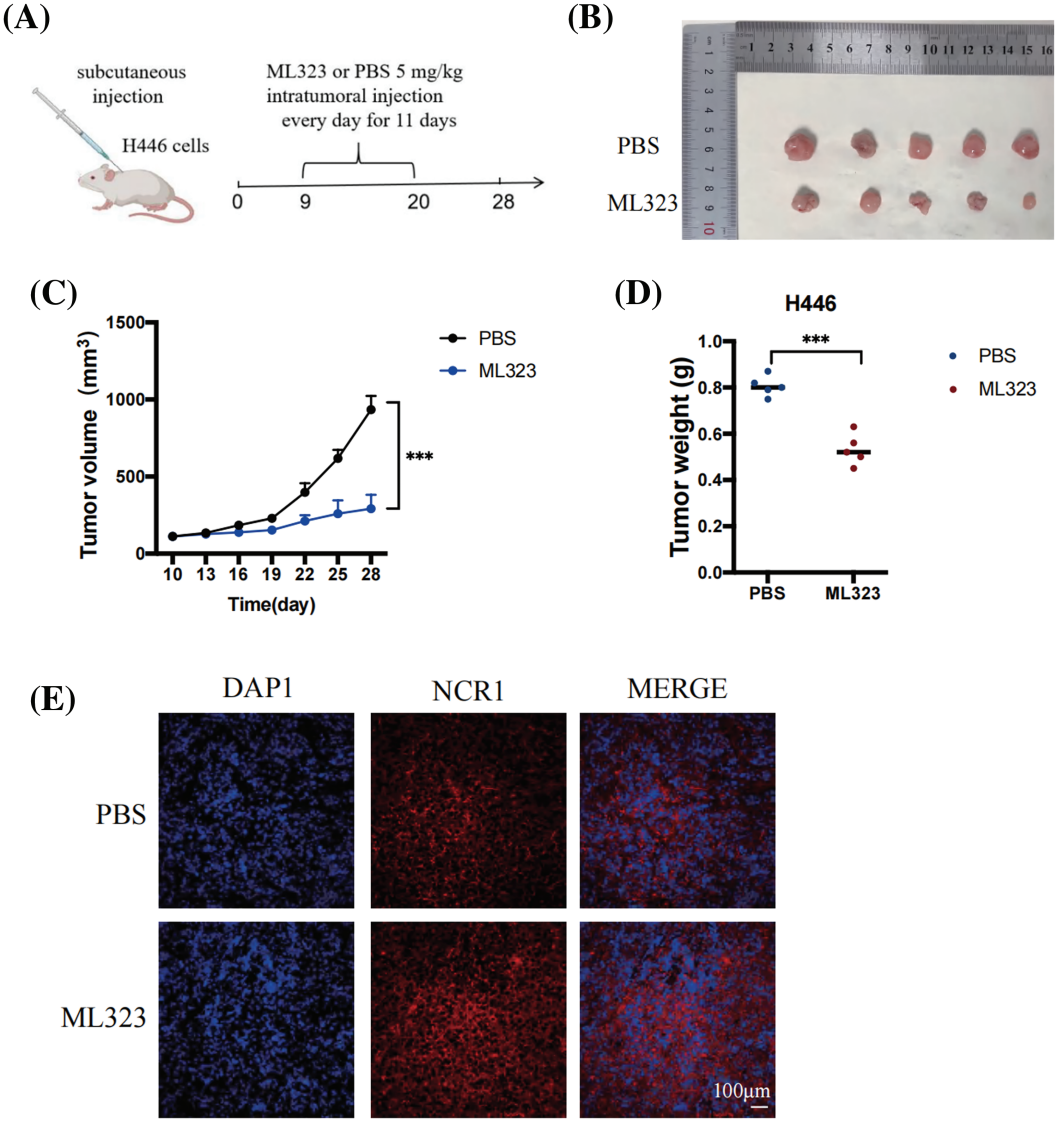
USP1 inhibition suppressed SCLC growth *in vivo*. (A) NCI-H446 cells (1 × 10^7^) were subcutaneously injected into athymic nude mice. Nine days after tumor transplantation, mice were intratumorally injected with either ML323 or PBS daily for 11 days. (B) Representative pictures depicting the excised subcutaneous tumors on day 28. (C) Subcutaneous tumor size was measured every 3 days using calipers. Tumor volume was calculated using the following formula: (length × width^2^)/2. The calculation was performed using mean and standard deviation. The unpaired *t*-test was used to perform statistical analysis (***p < 0.001). (D) Tumor weight was measured during harvest; the results were presented as mean ± standard deviation (unpaired *t*-test, ***p < 0.001). (E) Representative pictures of immunohistochemical staining of NCR1 in SCLC (Scale bar, 100 μm).

To determine tumor cell killing by NK cells, studies have identified athymic nude mice with robust NK cell function as appropriate models [25–27]. Therefore, we applied a nude-mice xenograft tumor model to further confirm the significance of USP1 inhibitors in SCLC. Nine days after tumor transplantation, ML323 or PBS were intratumorally injected into the mice daily for 11 days. After 28 days of transplantation, mice were euthanized, followed by the collection of xenograft tumors (Figs. 5A and 5B). Tumor size was assessed at intervals of 3 days. We observed that USP1 inhibition substantially impeded tumor growth (Figs. 5C and 5D). Immunohistochemical staining revealed increased expression of NCR1, a reliable marker for NK cells, in the USP1 inhibition group compared with the negative control group (Fig. 5E).

## Discussion

Considering the paucity of efficient molecular targets and the unfavorable overall outcomes observed in individuals with SCLC, performing investigations to identify prognostic biomarkers and therapeutic molecular targets is vital. In this study, we successfully identified USP1 as a prognostic marker for SCLC. USP1 was strongly associated with CD56^dim^ NK cell infiltration, and USP1 overexpression in SCLC cells suppressed NK cell-mediated cytotoxicity. These findings suggest that USP1 impedes NK cell-mediated antitumor immune responses, negatively affecting overall prognosis.

USP1 serves as a pivotal regulator of DNA repair responses, particularly in the Fanconi anemia pathway and DNA translation [11,12]. Moreover, USP1 can deubiquitinate and stabilize DNA-binding protein inhibitors [28], which play vital roles in various cancer-related processes, including cell differentiation, migration, invasion, and metastasis [29,30]. Consistent with our findings, USP1 levels are increased in osteosarcoma, colorectal cancer, non-small cell lung cancer, and gastric cancer [17,28,31,32]. Additionally, high USP1 expression is correlated with the unfavorable prognosis of renal cell carcinoma, ovarian cancer, and liver cancer [18,19,33]. Many research studies have revealed the role and mechanisms of USP1 in cancer growth and metastasis. USP1 is involved in various tumor-related activities, including controlling autophagy [24,34], developing chemotherapy resistance [35,36], promoting cell growth [37–39], and facilitating cellular movement [38,39].

Although researchers have acknowledged the essential role of USP1 in oncogenesis and metastasis, its precise function in SCLC remains unclear. Recent evidence has highlighted the vital function of USP1 in immune regulation, encompassing its role in controlling inflammation, promoting T helper type 17 cell differentiation, and inhibiting immunosuppressive regulatory T cell differentiation [14,15,40]. In the present study, we revealed that USP1 may inhibit NK cell-mediated immune responses in cancer cells. GSEA revealed that USP1 is negatively correlated with various NK cell-related pathways, including NK cell-mediated cytotoxicity, NK cell activation, and NK cell-mediated immunity. Furthermore, coculture experiments revealed that NK cells can efficiently kill USP1-depleted SCLC cells and that USP1 inhibition suppresses SCLC growth *in vivo*.

The effect of TILs on prognosis is different among various cancer types because of the diverse tumor microenvironment. Studies have revealed the correlation between TILs and clinical results for different cancer types [41–44]. To improve our understanding of the tumor microenvironment in SCLC, we investigated different immune cell populations and determined their effect on clinical outcomes. Our study findings suggest that a high-CD56^dim^ NK cell count can serve as a positive prognostic marker for SCLC. Two distinct human NK cell types are known: CD56^bright^ and CD56^dim^ subsets. CD56^dim^ NK cells can promote antitumor responses owing to their intrinsic cytotoxic capabilities [45]. In the present study, we discovered that increased levels of CD56^dim^ NK cells are associated with the improved survival of patients with SCLC, as revealed by both univariate and multivariate analyses. Using genetically modified mouse models, Best et al. [7] reported that the cytotoxic function of NK cells regulates SCLC metastasis. Interestingly, the absence of CD8^+^ T cells did not affect distant metastasis *in vivo* [7].

Considering the exceptional success of ICB therapy in cancer treatment, tumor evasion from T cell immunity and T cell-based immunotherapy has been the primary focus. However, our study underscores the importance of NK-cell-mediated immune response evasion in SCLC progression and growth. T cells can identify cancer cells by detecting altered or distinct peptide sequences presented by human leukocyte antigen (HLA); however, the overall response rate of ICBs is significantly low in patients with SCLC, despite the substantial mutational burden observed in these individuals. Decreased HLA expression may contribute to this limited response. In contrast, NK cells can be a compelling alternative for cancer immunotherapy because they can eliminate cancer cells without relying on neoantigens or HLA expression.

Animal models have demonstrated that NK cells can hinder tumor metastasis [46,47]. In clinical settings, NK cell activity is inversely correlated with cancer incidence [48,49]. On the other hand, NK cell infiltration into tumors is associated with a better prognosis in several cancer types [44,50,51]. In our study, we demonstrated that ML323 [19,24], a specific USP1 inhibitor, increases the susceptibility of SCLC cells to NK cell-mediated cytotoxicity *in vitro* and inhibits SCLC growth *in vivo*. ML323 was designed to selectively target USP1, exhibiting potent inhibitory activity with excellent selectivity over other deubiquitinases, proteases, and kinases [52]. Compared to previous USP1 inhibitors with promiscuous profiles, ML323 offers superior selectivity and limited cross-reactivity [52]. Its exceptional specificity towards USP1 minimizes off-target effects, ensuring the credibility and reliability of our results.

Collectively, our findings suggest that USP1 contributes to the poor prognosis of SCLC by suppressing NK cell-mediated antitumor immune responses. Considering its observed overexpression and crucial role in various aspects of cancer treatment, targeting USP1 emerges as a promising therapeutic strategy for SCLC. Notably, the ongoing phase I clinical trial (NCT05240898) is assessing the safety and effectiveness of KSQ-4279, a USP1 inhibitor, in patients with advanced cancer. USP1 plays a crucial role in DNA damage repair, immune regulation, and chemotherapy sensitization, making it an attractive target for SCLC treatment. However, it is important to recognize that the development and clinical translation of USP1 inhibitors pose certain challenges and risks. Achieving selectivity among the USP family members is a major challenge due to their high homology. To address this, Ongoing efforts aim to enhance selectivity against the entire USPs or DUBs family to minimize off-target effects. Another challenge lies in determining optimal dosing strategies and treatment regimens for USP1 inhibitors. The appropriate dosage and scheduling of these agents would need to be carefully established through preclinical studies and clinical trials to achieve maximum efficacy with minimal side effects.Furthermore, the potential benefits of targeting USP1 should also be considered. While our study primarily focuses on the role of NK cells in SCLC, it is important to consider the possible involvement of other immune cell populations in the tumor microenvironment. Investigating the interplay between USP1 and various immune cell populations, such as T cells, macrophages, and dendritic cells, would provide a more comprehensive understanding of the complex immune responses in SCLC. Overall, while targeting USP1 holds promise as a therapeutic strategy for SCLC, further research efforts are needed to fully comprehend their therapeutic potential and determine the optimal application in SCLC and other cancer types.

This study has several limitations. First, it is important to acknowledge that the study sample size was relatively small, which may limit the generalizability of the findings. Therefore, further validation with larger independent cohorts will be necessary to confirm and strengthen the observed correlations. Second, we acknowledge that the quantification of immune cell populations in our study was based on bulk tumor gene expression data. While this approach provides valuable insights into the overall immune landscape, it may not fully capture the cellular heterogeneity within the tumor microenvironment. To obtain more detailed insights, future studies utilizing single-cell RNA sequencing or other methods capable of capturing cellular heterogeneity are warranted. Third, although we demonstrated an association between increased USP1 expression and poor survival prognosis as well as NK cell evasion, it is important to acknowledge the limitations of inferring causation from observational data. Future research aimed at investigating the causal relationship between USP1, NK cells, and SCLC would be valuable for further understanding the underlying mechanisms. One potential direction for future research is the use of interventional trials or genetically engineered animal models to assess the effects of USP1 inhibition on NK cell function and SCLC progression. Additionally, the precise molecular mechanism by which USP1 inhibits NK cell-mediated immunity remains incompletely understood. Therefore, future investigations should focus on unraveling the specific pathways and molecular events through which USP1 influences NK cell responses.

In summary, USP1 is associated with the poor prognosis of SCLC possibly by inhibiting NK cell-mediated cytotoxicity against SCLC cells and inducing immune evasion. Additional investigation on the role of USP1 as a potential therapeutic target in SCLC is warranted and should be pursued in both preclinical and clinical studies.

## Supplementary Materials

**Supplementary Table 1:** Univariate Cox regression analysis of TILs in SCLC

	George’s study (n = 77)			GSE60052 dataset (n = 48)		
	HR	95% CI	*p*-value	HR	95% CI	*p*-value
Activated B cell	0.53	(0.082–3.4)	0.5	0.02	(0.00038–1)	0.052
Activated CD4 T cell	1.1	(0.051–23)	0.96	0.028	(6.1e−05–13)	0.25
Activated CD8 T cell	0.73	(0.09–5.9)	0.77	0.00035	(6.5e-07–0.19)	0.013
Activated dendritic cell	0.095	(0.00076–12)	0.34	0.009	(1.6e−05–5)	0.14
CD56^bright^ natural killer cell	850000	(13–5.7e + 10)	0.016	2.90E-06	(6.9e−10–0.012)	0.0028
**CD56**^**dim**^ natural killer cell^*^	**8.90E-07**	(7.6e−10–0.001)	**0.0001**	**0.00025**	(2.4e−07–0.26)	**0.019**
Central memory CD4 T cell	0.042	(0.00019–9.4)	0.25	0.05	(0.00016–16)	0.31
Central memory CD8 T cell	0.14	(0.00054–37)	0.49	0.019	(8.4e−05–4.4)	0.15
Effector memeory CD4 T cell	26000	(20–3.5e+07)	0.0055	1.6	(0.0076–330)	0.87
Effector memeory CD8 T cell	0.49	(0.038–6.2)	0.58	0.035	(8e−04–1.6)	0.084
Eosinophil	0.21	(0.0069-6.1)	0.36	0.71	(0.011–44)	0.87
Gamma delta T cell	13	(0.032–5400)	0.4	0.043	(1.1e−05–170)	0.46
Immature B cell	0.89	(0.12–6.7)	0.91	0.051	(0.0016–1.6)	0.091
Immature dendritic cell	17	(0.041–7000)	0.36	0.0092	(5.6e−06–15)	0.22
Macrophage	0.85	(0.03–24)	0.93	0.27	(0.0032–22)	0.56
Mast cell	1.2	(0.049–27)	0.93	0.36	(0.015–8.8)	0.53
MDSC	0.62	(0.1–3.8)	0.61	0.043	(0.0014–1.3)	0.071
Memory B cell	0.1	(0.00025–40)	0.45	0.36	(0.0014–97)	0.72
Monocyte	0.00013	(2.7e−09–5.9)	0.1	0.046	(8.1e−05–26)	0.34
Natural killer cell	0.039	(7.1e−05–21)	0.31	2.70E-08	(2.8e−13–0.0027)	0.003
Natural killer T cell	6.1	(0.022–1700)	0.53	0.01	(4.2e−05–2.4)	0.1
Neutrophil	16	(0.066–4100)	0.32	0.55	(0.035–8.6)	0.67
Plasmacytoid dendritic cell	0.084	(3.9e−05–180)	0.53	8.20E-06	(5.3e−10–0.13)	0.017
Regulatory T cell	0.53	(0.038–7.2)	0.63	0.077	(0.00091–6.5)	0.26
T follicular helper cell	0.89	(0.011-70)	0.96	0.0016	(2.6e−06–0.92)	0.047
Type 1 T helper cell	0.54	(0.0067–44)	0.78	0.0064	(1.9e−05–2.1)	0.088
Type 17 T helper cell	0.29	(0.00063–140)	0.7	0.0046	(1.2e−06–18)	0.2
Type 2 T helper cell	1	(0.00076–1400)	1	0.15	(1e−04–220)	0.61

Note: *CD56^dim^ NK cell abundance was significantly associated with patient survival in both George’s cohort and the GSE60052 cohort.

**Supplementary Table 2:** Sensitivity analysis of USP1 expression and overall survival at different cutoff values

Cutoff value (USP1 high expression)	Hazard ratio	95% confidence interval	*p*-value
>75%	2.1	1.1–3.9	0.019
>50%	2.2	1.2–3.9	0.011
>25%	5.2	2.2–13	<0.001

## Data Availability

The data generated for this study are available from the authors upon request.

## References

[1] Rudin, C. M., Brambilla, E., Faivre-Finn, C., Sage, J. (2021). Small-cell lung cancer. Nature Reviews Disease Primers*,* 7 *(*1*),* 3. 10.1038/s41572-020-00235-0; 33446664 PMC8177722

[2] Gazdar, A. F., Bunn, P. A., Minna, J. D. (2017). Small-cell lung cancer: What we know, what we need to know and the path forward. Nature Reviews Cancer*,* 17 *(*12*),* 725–737. 10.1038/nrc.2017.87; 29077690

[3] Ye, Z., Huang, Y., Ke, J., Zhu, X., Leng, S. et al. (2021). Breakthrough in targeted therapy for non-small cell lung cancer. Biomedicine & Pharmacotherapy*,* 133*,* 111079. 10.1016/j.biopha.2020.111079 33378976

[4] Horn, L., Mansfield, A. S., Szczesna, A., Havel, L., Krzakowski, M. et al. (2018). First-line atezolizumab plus chemotherapy in extensive-stage small-cell lung cancer. New England Journal of Medicine*,* 379 *(*23*),* 2220–2229. 10.1056/NEJMoa1809064; 30280641

[5] Ready, N., Farago, A. F., de Braud, F., Atmaca, A., Hellmann, M. D. et al. (2019). Third-line nivolumab monotherapy in recurrent SCLC: CheckMate 032. Journal of Thoracic Oncology*,* 14 *(*2*),* 237–244. 10.1016/j.jtho.2018.10.003; 30316010 PMC8050700

[6] Khalil, D. N., Smith, E. L., Brentjens, R. J., Wolchok, J. D. (2016). The future of cancer treatment: Immunomodulation, CARs and combination immunotherapy. Nature Reviews Clinical Oncology*,* 13 *(*5*),* 273–290. 10.1038/nrclinonc.2016.25; 26977780 PMC5551685

[7] Best, S. A., Hess, J. B., Souza-Fonseca-Guimaraes, F., Cursons, J., Kersbergen, A. et al. (2020). Harnessing natural killer immunity in metastatic SCLC. Journal of Thoracic Oncology*,* 15 *(*9*),* 1507–1521. 10.1016/j.jtho.2020.05.008; 32470639

[8] Zhu, M., Huang, Y., Bender, M. E., Girard, L., Kollipara, R. et al. (2021). Evasion of innate immunity contributes to small cell lung cancer progression and metastasis. Cancer Research*,* 81 *(*7*),* 1813–1826. 10.1158/0008-5472.CAN-20-2808; 33495232 PMC8137539

[9] George, J., Lim, J. S., Jang, S. J., Cun, Y., Ozretic, L. et al. (2015). Comprehensive genomic profiles of small cell lung cancer. Nature*,* 524 *(*7563*),* 47–53. 10.1038/nature14664; 26168399 PMC4861069

[10] Jiang, L., Huang, J., Higgs, B. W., Hu, Z., Xiao, Z. et al. (2016). Genomic landscape survey identifies SRSF1 as a key oncodriver in small cell lung cancer. PLoS Genetics*,* 12 *(*4*),* e1005895. 10.1371/journal.pgen.1005895; 27093186 PMC4836692

[11] Liang, F., Miller, A. S., Longerich, S., Tang, C., Maranon, D. et al. (2019). DNA requirement in FANCD2 deubiquitination by USP1-UAF1-RAD51AP1 in the Fanconi anemia DNA damage response. Nature Communications*,* 10 *(*1*),* 2849. 10.1038/s41467-019-10408-5; 31253762 PMC6599204

[12] Das, D. S., Das, A., Ray, A., Song, Y., Samur, M. K. et al. (2017). Blockade of deubiquitylating enzyme USP1 inhibits DNA repair and triggers apoptosis in multiple myeloma cells. Clinical Cancer Research*,* 23 *(*15*),* 4280–4289. 10.1158/1078-0432.CCR-16-2692; 28270494 PMC5540781

[13] Antonenko, S., Zavelevich, M., Telegeev, G. (2023). The role of USP1 deubiquitinase in the pathogenesis and therapy of cancer. Acta Biochimica Polonica*,* 70 *(*2*),* 219–231. 10.18388/abp.2020_6636; 37331010

[14] Omilusik, K. D., Nadjsombati, M. S., Yoshida, T. M., Shaw, L. A., Goulding, J. et al. (2021). Ubiquitin specific protease 1 expression and function in T cell immunity. Journal of Immunology*,* 207 *(*5*),* 1377–1387. 10.4049/jimmunol.2100303 PMC8387442 34380645

[15] Song, H., Zhao, C., Yu, Z., Li, Q., Yan, R. et al. (2020). UAF1 deubiquitinase complexes facilitate NLRP3 inflammasome activation by promoting NLRP3 expression. Nature Communications*,* 11 *(*1*),* 6042. 10.1038/s41467-020-19939-8; 33247121 PMC7695691

[16] Mussell, A., Shen, H., Chen, Y., Mastri, M., Eng, K. H. et al. (2020). USP1 regulates TAZ protein stability through ubiquitin modifications in breast cancer. Cancers*,* 12 *(*11*),* 3090. 10.3390/cancers12113090; 33114077 PMC7690829

[17] Garcia-Santisteban, I., Peters, G. J., Giovannetti, E., Rodriguez, J. A. (2013). USP1 deubiquitinase: Cellular functions, regulatory mechanisms and emerging potential as target in cancer therapy. Molecular Cancer*,* 12*,* 91. 10.1186/1476-4598-12-91; 23937906 PMC3750636

[18] Woo, S. M., Kim, S., Seo, S. U., Kim, S., Park, J. W. et al. (2022). Inhibition of USP1 enhances anticancer drugs-induced cancer cell death through downregulation of survivin and miR-216a-5p-mediated upregulation of DR5. Cell Death & Disease*,* 13 *(*9*),* 821. 10.1038/s41419-022-05271-0 36153316 PMC9509337

[19] Song, B., Jiang, Y., Jiang, Y., Lin, Y., Liu, J. (2022). ML323 suppresses the progression of ovarian cancer via regulating USP1-mediated cell cycle. Frontiers in Genetics*,* 13*,* 917481. 10.3389/fgene.2022.917481; 35923700 PMC9340375

[20] Ma, A., Tang, M., Zhang, L., Wang, B., Yang, Z. et al. (2019). USP1 inhibition destabilizes KPNA2 and suppresses breast cancer metastasis. Oncogene*,* 38 *(*13*),* 2405–2419. 10.1038/s41388-022-02215-y; 30531833

[21] Cai, L., Liu, H., Huang, F., Fujimoto, J., Girard, L. et al. (2021). Cell-autonomous immune gene expression is repressed in pulmonary neuroendocrine cells and small cell lung cancer. Communications Biology*,* 4 *(*1*),* 314. 10.1038/s42003-021-01842-7; 33750914 PMC7943563

[22] Subramanian, A., Tamayo, P., Mootha, V. K., Mukherjee, S., Ebert, B. L. et al. (2005). Gene set enrichment analysis: A knowledge-based approach for interpreting genome-wide expression profiles. Proceedings of the National Academy of Sciences of the United States of America*,* 102 *(*43*),* 15545–15550. 10.1073/pnas.0506580102.; 16199517 PMC1239896

[23] Bindea, G., Mlecnik, B., Tosolini, M., Kirilovsky, A., Waldner, M. et al. (2013). Spatiotemporal dynamics of intratumoral immune cells reveal the immune landscape in human cancer. Immunity*,* 39 *(*4*),* 782–795. 10.1016/j.immuni.2013.10.003; 24138885

[24] Sun, Y., Sha, B., Huang, W., Li, M., Zhao, S. et al. (2022). ML323, a USP1 inhibitor triggers cell cycle arrest, apoptosis and autophagy in esophageal squamous cell carcinoma cells. Apoptosis*,* 27 *(*7-8*),* 545–560. 10.1007/s10495-022-01736-x; 35654870

[25] Ashraf, Y., Mansouri, H., Laurent-Matha, V., Alcaraz, L. B., Roger, P. et al. (2019). Immunotherapy of triple-negative breast cancer with cathepsin D-targeting antibodies. Journal for ImmunoTherapy of Cancer*,* 7 *(*1*),* 29. 10.1186/s40425-019-0498-z.; 30717773 PMC6360707

[26] Malladi, S., Macalinao, D. G., Jin, X., He, L., Basnet, H. et al. (2016). Metastatic latency and immune evasion through autocrine inhibition of WNT. Cell*,* 165 *(*1*),* 45–60. 10.1016/j.cell.2016.02.025; 27015306 PMC4808520

[27] Elinav, E., Abd-Elnabi, A., Pappo, O., Bernstein, I., Klein, A. et al. (2006). Suppression of hepatocellular carcinoma growth in mice via leptin, is associated with inhibition of tumor cell growth and natural killer cell activation. Journal of Hepatology*,* 44 *(*3*),* 529–536. 10.1016/j.jhep.2005.08.013; 16310278

[28] Williams, S. A., Maecker, H. L., French, D. M., Liu, J., Gregg, A. et al. (2011). USP1 deubiquitinates ID proteins to preserve a mesenchymal stem cell program in osteosarcoma. Cell*,* 146 *(*6*),* 918–930. 10.1016/j.cell.2011.07.040; 21925315

[29] Perk, J., Iavarone, A., Benezra, R. (2005). Id family of helix-loop-helix proteins in cancer. Nature Reviews Cancer*,* 5 *(*8*),* 603–614. 10.1038/nrc1673; 16034366

[30] Yokota, Y., Mori, S. (2002). Role of Id family proteins in growth control. Journal of Cellular Physiology*,* 190 *(*1*),* 21–28. 10.1002/jcp.10042; 11807807

[31] Meng, D., Li, D. (2022). Ubiquitin-specific protease 1 overexpression indicates poor prognosis and promotes proliferation, migration, and invasion of gastric cancer cells. Tissue and Cell*,* 74*,* 101723. 10.1016/j.tice.2021.101723; 34990966

[32] Xu, X., Li, S., Cui, X., Han, K., Wang, J. et al. (2019). Inhibition of ubiquitin specific protease 1 sensitizes colorectal cancer cells to DNA-Damaging chemotherapeutics. Frontiers in Oncology*,* 9*,* 1406. 10.3389/fonc.2019.01406; 31921663 PMC6930197

[33] Zhao, Y., Xue, C., Xie, Z., Ouyang, X., Li, L. (2020). Comprehensive analysis of ubiquitin-specific protease 1 reveals its importance in hepatocellular carcinoma. Cell Proliferation*,* 53 *(*10*),* e12908. 10.1111/cpr.12908; 32951278 PMC7574869

[34] Raimondi, M., Cesselli, D., di Loreto, C., La Marra, F., Schneider, C. et al. (2019). USP1 (ubiquitin specific peptidase 1) targets ULK1 and regulates its cellular compartmentalization and autophagy. Autophagy*,* 15 *(*4*),* 613–630. 10.1080/15548627.2018.1535291; 30335599 PMC6526860

[35] Gong, H., Liu, L., Cui, L., Ma, H., Shen, L. (2021). ALKBH5-mediated m6A-demethylation of USP1 regulated T-cell acute lymphoblastic leukemia cell glucocorticoid resistance by Aurora B. Molecular Carcinogenesis*,* 60 *(*9*),* 644–657. 10.1002/mc.23330; 34169564

[36] Sonego, M., Pellarin, I., Costa, A., Vinciguerra, G. L. R., Coan, M. et al. (2019). USP1 links platinum resistance to cancer cell dissemination by regulating Snail stability. Science Advances*,* 5 *(*5*),* eaav3235. 10.1126/sciadv.aav3235; 31086816 PMC6506239

[37] Chen, Z., Ma, Y., Guo, Z., Song, D., Chen, Z. et al. (2022). Ubiquitin-specific protease 1 acts as an oncogene and promotes lenvatinib efficacy in hepatocellular carcinoma by stabilizing c-kit. Annals of Hepatology*,* 27 *(*2*),* 100669. 10.1016/j.aohep.2022.100669; 35045360

[38] Liu, S., Xiang, Y., Wang, B., Gao, C., Chen, Z. et al. (2023). USP1 promotes the aerobic glycolysis and progression of T-cell acute lymphoblastic leukemia via PLK1/LDHA axis. Blood Advances*,* 7 *(*13*),* 3099–3112. 10.1182/bloodadvances.2022008284; 36912760 PMC10362547

[39] Liao, Y., Shao, Z., Liu, Y., Xia, X., Deng, Y. et al. (2021). USP1-dependent RPS16 protein stability drives growth and metastasis of human hepatocellular carcinoma cells. Journal of Experimental and Clinical Cancer Research*,* 40 *(*1*),* 201. 10.1186/s13046-021-02008-3; 34154657 PMC8215741

[40] Zhu, X., Wang, P., Zhan, X., Zhang, Y., Sheng, J. et al. (2023). USP1-regulated reciprocal differentiation of Th17 cells and Treg cells by deubiquitinating and stabilizing TAZ. Cellular & Molecular Immunology*,* 20 *(*3*),* 252–263. 10.1038/s41423-022-00969-9 36600049 PMC9970968

[41] Autio, M., Leivonen, S. K., Bruck, O., Karjalainen-Lindsberg, M. L., Pellinen, T. et al. (2022). Clinical impact of immune cells and their spatial interactions in diffuse large B-cell lymphoma microenvironment. Clinical Cancer Research*,* 28 *(*4*),* 781–792. 10.1158/1078-0432.CCR-21-3140; 34907083 PMC9377736

[42] Wang, J., Li, R., Cao, Y., Gu, Y., Fang, H. et al. (2021). Intratumoral CXCR5^+^CD8^+^T associates with favorable clinical outcomes and immunogenic contexture in gastric cancer. Nature Communications*,* 12 *(*1*),* 3080. 10.1038/s41467-021-23356-w; 34035252 PMC8149695

[43] Valpione, S., Mundra, P. A., Galvani, E., Campana, L. G., Lorigan, P. et al. (2021). The T cell receptor repertoire of tumor infiltrating T cells is predictive and prognostic for cancer survival. Nature Communications*,* 12 *(*1*),* 4098. 10.1038/s41467-021-24343-x; 34215730 PMC8253860

[44] Melaiu, O., Chierici, M., Lucarini, V., Jurman, G., Conti, L. A. et al. (2020). Cellular and gene signatures of tumor-infiltrating dendritic cells and natural-killer cells predict prognosis of neuroblastoma. Nature Communications*,* 11 *(*1*),* 5992. 10.1038/s41467-020-19781-y; 33239635 PMC7689423

[45] Cozar, B., Greppi, M., Carpentier, S., Narni-Mancinelli, E., Chiossone, L. et al. (2021). Tumor-infiltrating natural killer cells. Cancer Discovery*,* 11 *(*1*),* 34–44. 10.1158/2159-8290.CD-20-0655; 33277307 PMC7611243

[46] Lehmann, J., Caduff, N., Krzywinska, E., Stierli, S., Salas-Bastos, A. et al. (2023). Escape from NK cell tumor surveillance by NGFR-induced lipid remodeling in melanoma. Science Advances*,* 9 *(*2*),* eadc8825. 10.1126/sciadv.adc8825; 36638181 PMC9839334

[47] Delconte, R. B., Kolesnik, T. B., Dagley, L. F., Rautela, J., Shi, W. et al. (2016). CIS is a potent checkpoint in NK cell-mediated tumor immunity. Nature Immunology*,* 17 *(*7*),* 816–824. 10.1038/ni.3470; 27213690

[48] Imai, K., Matsuyama, S., Miyake, S., Suga, K., Nakachi, K. (2000). Natural cytotoxic activity of peripheral-blood lymphocytes and cancer incidence: An 11-year follow-up study of a general population. Lancet*,* 356 *(*9244*),* 1795–1799. 10.1016/S0140-6736(00)03231-1; 11117911

[49] Habif, G., Crinier, A., Andre, P., Vivier, E., Narni-Mancinelli, E. (2019). Targeting natural killer cells in solid tumors. Cellular & Molecular Immunology*,* 16 *(*5*),* 415–422. 10.1038/s41423-019-0224-2 30911118 PMC6474204

[50] Cursons, J., Souza-Fonseca-Guimaraes, F., Foroutan, M., Anderson, A., Hollande, F. et al. (2019). A gene signature predicting natural killer cell infiltration and improved survival in melanoma patients. Cancer Immunology Research*,* 7 *(*7*),* 1162–1174. 10.1158/2326-6066.CIR-18-0500; 31088844

[51] Bruno, A., Ferlazzo, G., Albini, A., Noonan, D. M. (2014). A think tank of TINK/TANKs: Tumor-infiltrating/tumor-associated natural killer cells in tumor progression and angiogenesis. Journal of the National Cancer Institute*,* 106 *(*8*),* dju200. 10.1093/jnci/dju200; 25178695 PMC4344546

[52] Dexheimer, T. S., Rosenthal, A. S., Luci, D. K., Liang, Q., Villamil, M. A. et al. (2014). Synthesis and structure-activity relationship studies of N-benzyl-2-phenylpyrimidin-4-amine derivatives as potent USP1/UAF1 deubiquitinase inhibitors with anticancer activity against nonsmall cell lung cancer. Journal of Medicinal Chemistry*,* 57*,* 8099–8110. 10.1021/jm5010495; 25229643 PMC4191588

